# Filiform Polyposis Secondary to Colonic Tuberculosis Presenting as Acute Colo-Colonic Intussusception

**DOI:** 10.1155/2015/578263

**Published:** 2015-05-31

**Authors:** Jacob S. Heng, Alan Baird, Marco R. Novelli, Robert N. Davidson, Rajinder P. Bhutiani

**Affiliations:** ^1^Department of General Surgery, Northwick Park Hospital, Harrow, London HA1 3UJ, UK; ^2^Imperial College London Faculty of Medicine, London SW7 2AZ, UK; ^3^Department of Pathology, St. Mark's Hospital, Harrow, London HA1 3UJ, UK; ^4^Department of Pathology, University College Hospital, London NW1 2BU, UK; ^5^Department of Infectious Diseases, Northwick Park Hospital, Harrow, London HA1 3UJ, UK

## Abstract

Filiform polyposis represents a rare but recognised manifestation on the varied spectrum of histopathology in colonic tuberculosis. We report a case of filiform polyposis secondary to colonic tuberculosis presenting as colo-colonic intussusception diagnosed on an abdominal computed tomography (CT) scan. The patient required urgent hemicolectomy and defunctioning ileostomy. Examination of the resected bowel lesions revealed filiform polyposis. Induced sputum samples from the patient grew *Mycobacterium tuberculosis*. The patient recovered well from the surgery and received treatment for tuberculosis. At last follow-up, he was awaiting the reversal of his ileostomy. The protean nature of histological findings in colonic tuberculosis and other current diagnostic challenges are discussed. The importance of maintaining a high index of suspicion for colonic tuberculosis and instituting early treatment is highlighted in this case.

## 1. Introduction

Diagnosing colonic tuberculosis may present many challenges in practice. In general, gastrointestinal tuberculosis may have wide variation in presenting complaint, pattern of distribution of lesions, gross macroscopic appearance of lesions, and histological findings [1, 2]. Bowel intussusception is a rare presentation of tuberculosis and usually the result of an associated inflammatory mass [3]. In adults, this may initially be confused for a neoplastic lesion [3]. Furthermore, histological findings frequently do not show the presence of mycobacteria or the classical “caseating granulomas” and often represent nonspecific inflammatory changes [1]. Such inflammatory changes are also seen in a range of inflammatory bowel diseases [4] and include filiform polyposis [5]. We present a case of filiform polyposis secondary to colonic tuberculosis presenting as colo-colonic intussusception.

## 2. Case Presentation

A 44-year-old previously fit and well Caucasian man presented with a one-day history of severe, unremitting, and diffuse colicky abdominal pain with absolute constipation. He had a four-month history of similar but less severe pain, preceded by loose stools with mucus and frequent PR bleeds on a background of poor appetite and 7 kg weight loss over six months. Family history was notable for colorectal cancer in his uncle at 71 years of age. Social history was significant for 10 cigarettes a day, 70 units of alcohol a week, and extensive travel history to Thailand. On examination, the lower abdomen was distended and diffusely tender with tinkling bowel sounds but no guarding or rigidity.

**Figure 1:**
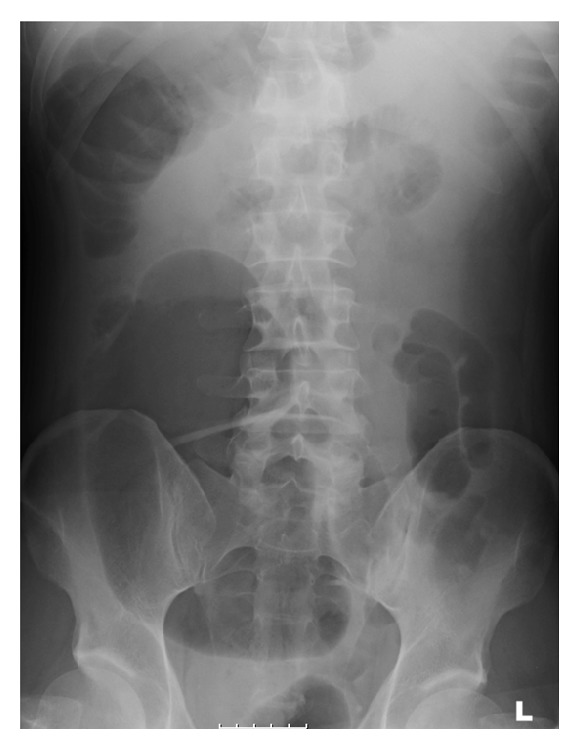
Abdominal X-ray at presentation showing hugely dilated caecum and transverse colon with paucity of gas in the descending colon.

**Figure 2:**
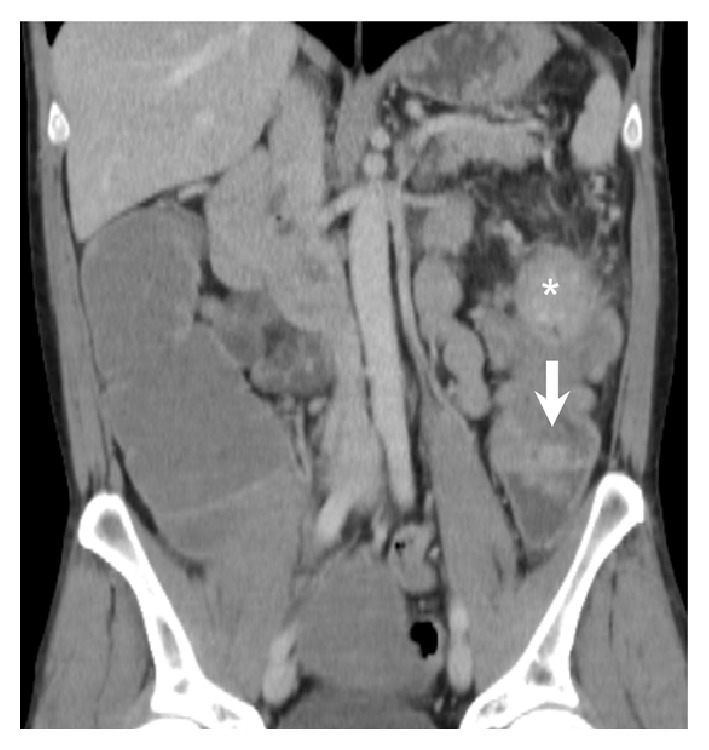
CT scan of abdomen (coronal section) showing intussusception of a polypoid lead point (*∗*) into the distal descending colon (arrow indicates direction of intussusception).

**Figure 3:**
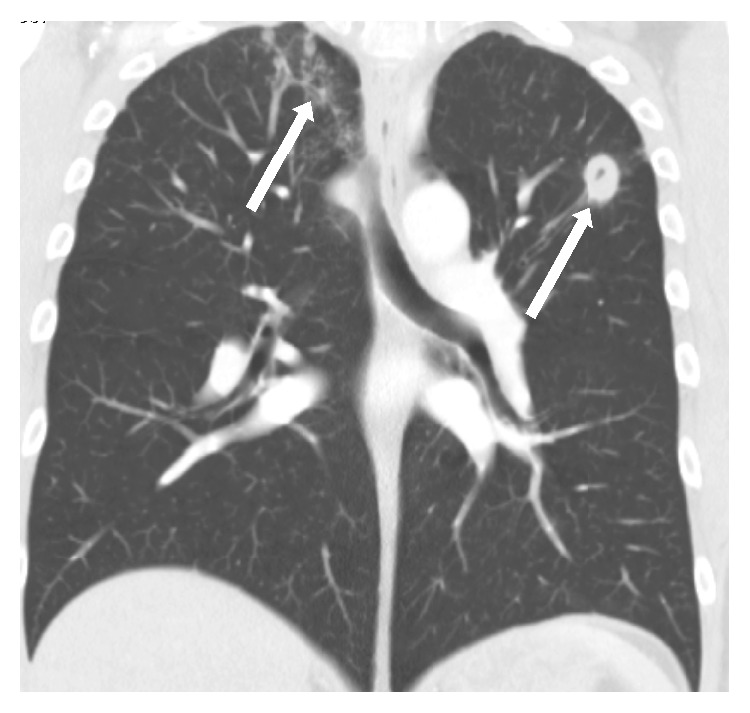
CT scan of chest (coronal section) showing bilateral cavitating lesions in the lung apices (indicated by arrows).

A plain supine abdominal radiograph showed a hugely dilated caecum (maximum diameter: 15 cm) and transverse colon (maximum diameter: 10 cm) with paucity of gas in the descending colon (Figure 1). A CT scan revealed apparent intussusception of a polypoid lead point into the distal descending colon (Figure 2) but no signs of bowel perforation as well as bilateral cavitating lesions in the lung apices (Figure 3).

An urgent exploratory laparotomy revealed an irregular mass in the distal transverse colon and another adjacent mass in the splenic flexure with apparent spontaneous resolution of the intussusception seen on CT. Widespread lymphadenopathy was noted in the transverse colon mesentery. A left hemicolectomy was undertaken with excision of the left-sided omentum, followed by a side-to-side anastomosis with a defunctioning loop ileostomy.

A Mantoux test yielded 16 mm induration, albeit in the context of prior BCG vaccination 30 years ago. However, alcohol and acid-fast bacilli (AAFB) were not detected in sputum samples. HIV test was negative.

**Figure 4:**
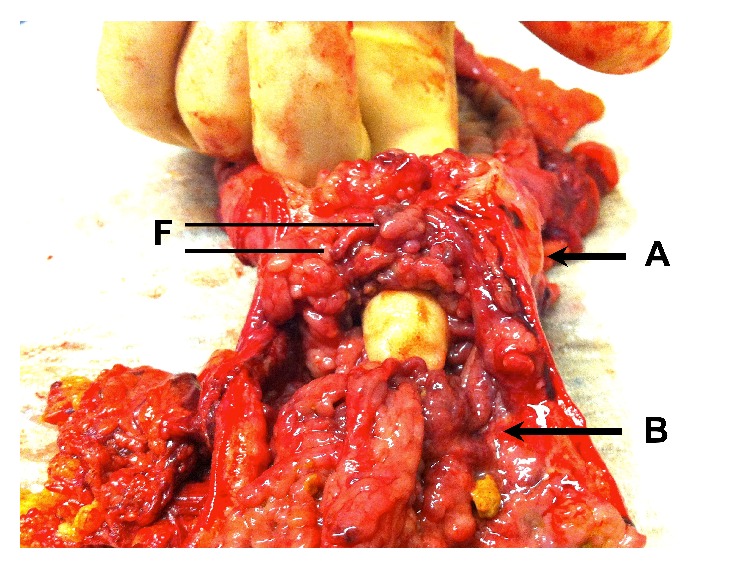
Section from left hemicolectomy showing a constricting mass in the distal transverse colon (Label A) and an adjacent synchronous mass in the proximal descending colon (Label B). Filiform polyps (F) are seen within both lesions.

**Figure 5:**
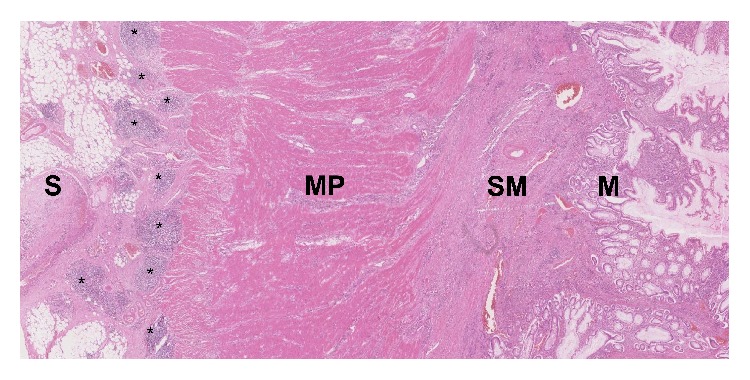
H&E histology slide 50x magnification showing subserosal lymphoid aggregates in a rosary pattern. S = serosa, *∗*  = lymphoid aggregate, MP = muscularis propria, SM = submucosa, and M = mucosa.

Histology sections from the colonic lesion showed an area of florid filiform polyposis (Figure 4) with no evidence of dysplasia but with adjacent ulceration of the colonic mucosa. The background colon showed areas of transmural chronic inflammation in the form of subserosal lymphoid aggregates arranged in a rosary pattern (Figure 5). No AAFB, granulomas, features of colitis, or diverticular disease were identified. Multiple lymph nodes examined showed reactive-type changes only. The appearances were most in keeping with localised filiform polyposis.

Despite the absence of AAFB on sputum microscopy and colonic histology, the Infectious Disease (ID) team treated the patient empirically for tuberculosis on a 6-month course of rifampicin (10 mg/kg/day up to 600 mg/day), isoniazid (5 mg/kg/day up to 300 mg/day), pyrazinamide (30 mg/kg/day up to 2 g/day), and ethambutol (15 mg/kg/day) on the basis of the cavitating lung lesions and positive Mantoux test. The patient recovered and was discharged from hospital a week later.

Serial sputum cultures eventually grew fully sensitive* Mycobacterium tuberculosis*. A follow-up CT scan of the chest three months later showed mild improvement of his lung lesions. A water-soluble contrast enema four months after surgery showed the large bowel to have no abnormalities. At the time of last follow-up six months after surgery, the patient was well and awaiting the reversal of his ileostomy. The patient was under close follow-up from his General Practitioner in close liaison with the Infectious Diseases team. Contact tracing was difficult for this patient as he traveled overseas frequently and was likely to have contracted tuberculosis while being overseas.

## 3. Discussion

Filiform polyposis is a rare entity that is most often encountered in the colon of patients with a history of inflammatory bowel disease (IBD) [6] and could be considered a variant of inflammatory pseudopolyps. Filiform polyposis is characterised by a large number of worm-like or finger-like polyps that can be localised and/or associated with benign strictures or generalised involving the whole colon [7]. The mucosa overlying the polyps is typically normal but may contain features of nonspecific acute or chronic inflammation, and is almost never dysplastic [4, 7]. The pathogenesis of filiform polyposis is not well understood, but it may represent an unregulated attempt at tissue repair following mucosal damage [4]. It is generally considered a nonspecific sequelae of diffuse mucosal inflammation [4]. Spark [8] postulated that such polyposis may be formed by a narrow band of submucosa being stimulated to proliferate by two closely adjacent, sharply demarcated, intensely inflamed and intermittently ulcerated zones, consistent with our case. To our knowledge, there has only been one reported case of colonic filiform polyposis associated with systemic tuberculosis to date [5].

Diagnosing colonic tuberculosis may present many challenges in practice. Colonic tuberculosis can often mimic IBD, in particular Crohn's disease. Macroscopically, colonic tuberculosis can result in IBD-like lesions such as segmental ulcers, generalised colitis, mucosal nodules, polyps, strictures, perforation, and fistulae [2]. Moreover, like Crohn's disease, colonic tuberculosis usually has a segmental pattern of distribution, involving two or more colonic segments in up to 58% of cases, with frequent concurrent involvement of the ileocaecal region [1]. Histology and ultimately microbiological culture of lesions may help to clarify the diagnosis. If there is no urgent indication for surgery, unlike in the case of our patient, colonoscopy may be a useful tool with diagnostic yield of up to 80% in patients with suspected colonic tuberculosis [1] by providing a means of obtaining biopsy samples for histology and culture in addition to examining the pattern of distribution and gross appearance of colonic lesions. It is worth noting that bowel intussusception due to tuberculosis, an acute indication for surgery, is a rare occurrence [3] and, to our knowledge, there has been no previously reported case of colo-colonic intussusception due to tuberculosis.

The histopathology of colonic tuberculosis may, however, represent a varied spectrum rather than a well-defined entity. Multiple biopsy samples may confirm granulomas that are typically located in the submucosa [1]. However, granulomas have been identified in only 41–48%, whereas pathognomonic caseating granulomas are found in only 19–38% of biopsies [1]. Indeed, in some cases of colonic tuberculosis, only nonspecific chronic inflammatory changes are seen without granulomas or caseation [9], such as in our case, and, as mentioned above, filiform polyposis has also been described in a single case [5]. Similarly, the culture of biopsy samples yields positive results in only 6–69% of patients [1] and typically takes at least 6 weeks to yield growth of mycobacteria. Furthermore, AAFBs may not be seen in the initial biopsy samples [10], especially if patients are immunocompetent and have had prior BCG vaccination [11], as in the case of our patient.

It is important to note that prior BCG vaccination in childhood does not preclude the development of subsequent tuberculosis. In a meta-analysis of BCG vaccine trials, BCG vaccination showed an overall 86% efficacy in protecting against miliary and meningeal tuberculosis and only heterogeneous efficacy against pulmonary tuberculosis [12], the most common adult form of the disease. Furthermore, the efficacy of the BCG vaccine had been shown to wane to an overall average of 14% after 10 years in a quantitative analysis of ten trials [12].

In our patient, his travel history, cavitating pulmonary lesions, and multiple segmental colonic involvement were all suggestive of tuberculosis. Although his colonic histology showed nonspecific inflammatory features with absence of AAFBs and granulomas,* Mycobacterium* grown in his sputum samples in his clinical context confirmed the diagnosis of colonic tuberculosis.

In conclusion, this case illustrates filiform polyposis as a rare but recognised manifestation on the varied spectrum of histopathology in colonic tuberculosis. This case also highlights the difficulties in diagnosing colonic tuberculosis and the importance of initiating early anti-tuberculous treatment in patients with a high index of suspicion for tuberculosis despite negative histology and/or microbiological culture.
